# Strengths and Weaknesses of Global Positioning System (GPS) Data-Loggers and Semi-structured Interviews for Capturing Fine-scale Human Mobility: Findings from Iquitos, Peru

**DOI:** 10.1371/journal.pntd.0002888

**Published:** 2014-06-12

**Authors:** Valerie A. Paz-Soldan, Robert C. Reiner, Amy C. Morrison, Steven T. Stoddard, Uriel Kitron, Thomas W. Scott, John P. Elder, Eric S. Halsey, Tadeusz J. Kochel, Helvio Astete, Gonzalo M. Vazquez-Prokopec

**Affiliations:** 1 Global Health Systems and Development Department, Tulane University School of Public Health and Tropical Medicine, New Orleans, Louisiana, United States of America; 2 Department of Entomology, University of California, Davis, Davis, California, United States of America; 3 Fogarty International Center, National Institutes of Health, Bethesda, Maryland, United States of America; 4 Graduate School of Public Health, San Diego State University, San Diego, California, United States of America; 5 Department of Environmental Studies, Emory University, Atlanta, Georgia, United States of America; 6 U.S. Navy Medical Research Unit No. 6, Iquitos, Peru; University of Queensland, Australia

## Abstract

Quantifying human mobility has significant consequences for studying physical activity, exposure to pathogens, and generating more realistic infectious disease models. Location-aware technologies such as Global Positioning System (GPS)-enabled devices are used increasingly as a gold standard for mobility research. The main goal of this observational study was to compare and contrast the information obtained through GPS and semi-structured interviews (SSI) to assess issues affecting data quality and, ultimately, our ability to measure fine-scale human mobility. A total of 160 individuals, ages 7 to 74, from Iquitos, Peru, were tracked using GPS data-loggers for 14 days and later interviewed using the SSI about places they visited while tracked. A total of 2,047 and 886 places were reported in the SSI and identified by GPS, respectively. Differences in the concordance between methods occurred by location type, distance threshold (within a given radius to be considered a match) selected, GPS data collection frequency (i.e., 30, 90 or 150 seconds) and number of GPS points near the SSI place considered to define a match. Both methods had perfect concordance identifying each participant's house, followed by 80–100% concordance for identifying schools and lodgings, and 50–80% concordance for residences and commercial and religious locations. As the distance threshold selected increased, the concordance between SSI and raw GPS data increased (beyond 20 meters most locations reached their maximum concordance). Processing raw GPS data using a signal-clustering algorithm decreased overall concordance to 14.3%. The most common causes of discordance as described by a sub-sample (n = 101) with whom we followed-up were GPS units being accidentally off (30%), forgetting or purposely not taking the units when leaving home (24.8%), possible barriers to the signal (4.7%) and leaving units home to recharge (4.6%). We provide a quantitative assessment of the strengths and weaknesses of both methods for capturing fine-scale human mobility.

## Author Summary

Being able to quantify human movement is important for studying activity patterns, exposure to pathogens and developing realistic infectious disease models. We compared fine-scale human mobility data obtained by Global Positioning System (GPS)-enabled devices and semi-structured interviews (SSI) from 160 individuals in Iquitos, Peru, in order to assess the quality of data using these two different approaches and our ability to measure fine-scale human mobility patterns in a resource-poor urban environment. Using various methods to process the GPS data, we found the SSI identified more locations a person had visited than GPS. Though the GPS gave more precise data, there were behavioral, technical, and analytical barriers. The SSI provided richer context and was easier to process, but also had more false positives. SSI was the only option for identifying locations retrospectively.

## Introduction

Knowledge of daily and routine individual human mobility patterns within urban settings are important for urban planning [1]–[3], developing transportation models [3], promoting healthy lifestyles [4], and understanding infectious disease dynamics [5]–[13]. Measuring mobility at fine spatial and temporal scales through classic data collection methods (e.g., interviews, diaries, direct observations) presents significant challenges, such as marked heterogeneities in the ability of individuals to recall the locations they visit, changes in people's lives that affect their daily mobility (e.g., new partners, change of jobs, school vacation) as well as privacy issues [11], [14]. These challenges can be exacerbated in resource-poor settings [6], [7], [10], [15], [16], such as our study site in Iquitos, Peru, due to the lack of complete and updated address maps (affecting geo-coding of self-reported addresses) and limitations in spatial literacy of interviewed individuals [11]. There is an urgent need to develop and validate easily deployable and culturally-sensitive tools that characterize a person's routine mobility in order to link such information to health outcomes [6], [10], [13], [17], [18]. This is of particular relevance for understanding infectious disease dynamics, given the dominant role mobility has in driving infectious contacts and thus pathogen transmission, emergence, persistence and propagation [5], [6], [8]–[13], [18]–[22].

The wide availability of emerging location-aware technologies such as Global Positioning System (GPS)-phones or data-loggers provides new opportunities to quantify human mobility at fine spatial and temporal scales. Their use in research projects is feasible: they have decreased in cost and size, the technology has improved (i.e., GPS chipsets are more efficient in acquiring and fixing a signal as well as in power consumption) and the units are widely accepted by study populations [6], [10], . Over the past ten years, GPS tracking (often coupled with other sensors) has taken a prominent role in physical activity and exposure research [17], [26], [27]. Their implementation, however, in infectious disease research has been limited in part due to the challenges in linking the positional data generated by such sensors with temporally and spatially discrete locations (i.e., a person's home) where pathogen exposure occurred, and more importantly, the complexities associated with the analysis of the vast amount of data that these sensors can generate. A recent systematic review [17] shows that most studies using GPS to track physical activity involve few participants (<20), track individuals over short time periods (<12 days) and are focused on specific age groups (children vs. adults) or environmental correlates of activity (e.g., park vs. school movement) [17], [27]. GPS-based tracking presents enormous opportunities for improving our understanding of individual space-time activities and how they influence health outcomes, which has been done in various studies [6], [7], [10], [11], [15], [16], [28].

GPS technology, however, also has limitations that need to be addressed before considering it a “gold standard” for mobility research [26]. Rates of GPS data loss can reach 92% due to signal drop-outs, dead batteries, participants not wearing the units, signal loss during the initialization period or misuse of the device [17]. In Stothard et al.'s study in Uganda, the authors found that the track logs of the small, wearable GPS units (i-gotU) were accurate compared to a more sophisticated and costly unit (Garmin Oregon 550t) – discordance of <7 m for the 15 households tested – but there was GPS malfunction in units that was believed to be related to “insufficiently robust hardware for field conditions” possibly due to humidity or quality of the software [10].

As part of a larger study investigating risk for dengue (a human disease caused by a mosquito transmitted virus) in Iquitos, Peru, we simultaneously implemented two methods to capture fine-scale human mobility patterns: GPS data-loggers and semi-structured interviews (SSI). Dengue is a mosquito-transmitted viral disease of humans in tropical and subtropical regions of the world that is a rapidly growing public health problem [29], [30]. The main goal of this observational study was to compare and contrast the information obtained through these two methods to assess the issues affecting data quality, and identify strengths and weaknesses of each approach. We used two methods to analyze GPS data, and compared GPS results obtained via both methods with the results from the SSI.

## Methods

### Study Setting

Our study took place in Iquitos, a large and geographically isolated city in the Amazon Basin of northeastern Peru that is accessible only by boat or plane [31], between September 2008 and August 2010. The city of Iquitos has a high population density (∼390,000 inhabitants), and a very informal and dynamic economic structure (33.4% of those economically active are either unemployed or informally employed) [31]. As observed in other resource-poor cities, Iquitos lacks a unified and updated address system. Car access and public transportation are limited and residents rely on personal motorcycles, ∼20,000 motorized rickshaws [“moto-taxis”], and a few bus lines to move throughout the city. The major industries in the area are small commercial enterprises, fishing, oil, lumber, tourism, and agriculture [7]. Iquitos is the home-base of an extensive, ongoing, long-term project since 1999 led by the University of California at Davis/U.S. Naval Medical Research Unit 6-Iquitos group [5]–[7], [15], [16], [32] studying the environmental, entomologic, epidemiologic and behavioral determinants of dengue virus transmission.

### Instruments

Two methods for obtaining fine-scale human mobility data were simultaneously implemented: (1) GPS data-loggers (“i-gotU GT120”, Mobile Action Technology Inc.) and (2) semi-structured interviews (SSIs).

Descriptions of GPS features, spatial accuracy, acceptance by participants and device deployment associated to this study were reported previously [11], [12]. The main attributes of selected units were: (1) data storage capacity and battery life capable of recording at least 3 days of data; (2) high spatial accuracy (∼4–10 m); (3) durable, water resistant and tamper-proof; (4) light weight (<50 g); (5) carrying mechanism (lanyard around neck) widely accepted by participants of different ages/sex; (6) little to no maintenance required by study participants; (7) low cost ($49); and (8) password protection and a special socket for data download (to protect participant's confidentiality). The units are easily worn on a neck strap or in a pocket, and have been used to track routine movement patterns of Iquitos residents over the past three years with a high level of acceptance (98%) [16].

Based on the known limitations of classic interview instruments to capture overt behaviors in space and time [2], [33]–[35], and guided by findings from focus group discussions performed in Iquitos [16], we designed a SSI for capturing positional and temporal information of routine human mobility. Key findings from the focus groups that guided the survey development included [16]: (1) people could clearly identify many of the routine locations they visited, although they sometimes needed certain “triggers” for recall (and these were identified), (2) there were marked differences in reported mobility routines by gender and age groups; and (3) there were clear “common activity spaces” (markets, recreational spots, etc). The developed SSI contained one section listing commonly visited locations, such as markets, health facilities, and schools, and a section that used field-tested triggers to help people recall “individual” locations visited (such as relatives' houses) in the last 14 days. Participants also gave estimates of time spent in each location per week. High resolution satellite (Quickbird, Digitalglobe, CO) and digitized street maps were used during the interview to prompt recall and to mark the position of the places mentioned.

### Recruitment, Participants, and Study Design

Participant recruitment was not random: we used purposive sampling and focused on two Iquitos neighborhoods participating in an ongoing longitudinal study on dengue epidemiology [32], [36], seeking a balanced number of males and females representing age ranges between 7 and 74 (see Table 1). We only excluded those who planned to spend more than a day outside of Iquitos during the following 14 days. Recruitment was performed by trained local technicians who provided a description of the study together with a pamphlet with specific information about the GPS units and the study in general [16]. In the first phase, conducted between September 2008 and March 2009, 59 participants were asked to use the GPS units at all times for a period of 14 days and respond to the SSI on day 15 asking for all the places they visited while GPS-tracked during those 14 previous days. The research team scheduled an exchange of the GPS units every three days to download data, verify function, and recharge batteries. At the time of GPS unit exchange, participants were asked about their experiences with the GPS, whether they had used it, if it had been forgotten and, if so, on what days. GPS units were programmed to track a person's position (latitude, longitude and time stamp) every 150 seconds. The second phase was conducted in July and August of 2010 with 101 participants, who were asked to follow the same procedures as before; use the GPS unit for 14 days and respond to the SSI on day 15. One component was added in this phase: within 3 days of data collection, survey data was entered into a database and GPS-collected data was processed so that information on the locations identified as visited by each method were overlaid in a Geographic Information System (ArcGIS 10, ESRI). With a series of maps noting the position of each place visited by either method, field technicians returned to the participants within 4–5 days to ask them about any discordant information (i.e., locations on the survey, but not registered on the GPS or vice versa). For Phase 2, the GPS collection frequency was increased to every 15 seconds (45 participants) and 90 seconds (56 participants) to assess the impact of data collection frequencies on GPS-SSI concordance. Whereas with 150 second programming, we could collect and recharge GPS units every 3 days, individuals wearing GPS units programmed at 15 and 90 seconds were provided with a charger and asked to charge the units daily because of the reduction in battery life. Our sample size was sufficient for a descriptive analysis and was limited due to intense participant follow-up for ∼20 days; i.e., recruiting and consenting, distributing GPS units, exchanging charged GPS units and collecting ones losing power, interviewing participants with SSI at day 14, geocoding locations immediately, inputting all data from GPS and SSI to overlay in a GIS, returning to participants for follow up interview. Considering these complexities, participant recruitment was limited to what was logistically feasible for our field teams.

**Table 1:** Demographic description of 160 participants for which concurrent semi-structured interviews and GPS tracking were performed.

	Number of participants		
	Phase 1 (n = 59)	Phase 2 (n = 101)	Total (n = 160)
**Sex**			
Male	52.5 (31)	34.7 (35)	41.3 (66)
Female	47.5 (28)	65.3 (66)	58.8 (94)
**Age structure**			
7–18	5.1 (3)	42.6 (43)	28.8 (46)
19–30	27.1 (16)	15.8 (16)	20.0 (32)
31–40	23.7 (14)	10.9 (11)	15.6 (25)
41–50	23.7 (14)	16.8 (17)	19.4 (31)
>50	20.3 (12)	13.9 (14)	16.3 (26)

Phase 1 included individuals who used the GPS units at all times for 14 days and responded (on day 15) to a retrospective SSI, whereas Phase 2 included the same methods as Phase 1, but in addition individuals were interviewed on day 18 about any discordant information (i.e., locations on the SSI but not registered on the GPS, or vice versa).

### Data Processing and Analysis

All locations reported on the SSI were identified in the Iquitos GIS and received a unique location code with geographic coordinates that link directly to a SQL database containing participant information. If the location was not already in our system or if there were doubts about the specific location, a research team member went to the described place to assign a geo-code. Based on geo-referenced city-block maps (courtesy of the Peruvian Navy) and field sketch maps, geo-referenced aerial photographs and high resolution satellite imagery (Quickbird, Digitalglobe, CO), a total of 48,365 Iquitos lots were digitized prior to initiation of this study. Given the lack of a formal and consistent address system, we assigned a unique code to each lot. A local GIS specialist on our research team updates the maps on a regular basis, making the Iquitos GIS one of the most complete and up to date geo-spatial databases generated for a resource-poor city of its size.

To obtain locations recorded by GPS units, the raw data was processed using an agglomerative algorithm (i-Cluster [15]). In simple terms, when GPS raw data was plotted over a satellite image of the city, we observed “clouds” over specific locations that were frequented by an individual [15]. These “clouds” mark locations that are the product of the frequency of going to that place and the time spent there. This data reduction algorithm works by aggregating consecutive GPS readings that are within a spatial (*d*) and temporal (*t*) window, and estimating the total time a participant spent within such a spatio-temporal buffer [15]. The algorithm also allows for identification of locations intermittently visited by applying a threshold time (*tintv*) in between visits. Based on the inherent spatial error of GPS data (e.g., 5–10 m) we determined the following configuration: *d* = 20 m, *t* = 15 min and *tintv* = 30 min, for tracking Iquitos participants. The resulting place derived from the i-Cluster algorithm was then manually assigned the nearest location ID in the Iquitos GIS.

For the analysis, we directly compared the raw GPS data to the SSI data. Because we know the exact GPS coordinates of every location reported in the SSI data, we could test to see how frequently the GPS unit reported that the individual was in the vicinity of each location. Specifically, for every participant and every location they visited, we calculated the distance from every GPS point registered for that participant to that location. For many locations, we have not just the location, but the footprint of the structure as a polygon within the Iquitos GIS. As such, we could calculate the distance from each GPS point to the boundary of each location (taking GPS points that were within the polygon to have distance zero from the structure). For both locations that we have the footprint of the structure and those that we just have a single GPS location representing the centroid of the building, we consider the location “visited” if there are a sufficient number of raw GPS points within a certain threshold distance of the location. We then vary the number of raw GPS points deemed sufficient (here we used 1, 5, and 10 points), as well as the distance threshold selected (defined as the distance allowed for what constitutes a “match” between locations recorded in the SSI compared to a nearby GPS point, in this study, ranging from 0 to 100 meters), to investigate the sensitivity of visitation.

We quantified the concordance between SSI and GPS in identifying places visited by participants by comparing the interview locations with (1) i-Cluster-derived locations and (2) raw GPS positions. To compare the interview with the i-Cluster inferred locations we mapped the locations identified by each method in a GIS (ArcMap 9.3; ESRI). Locations identified both by the GPS and the SSI were considered “concordant” and did not require follow up. All locations that were captured by either GPS or the SSI, but not both, were considered “discordant” and a research assistant was sent back to the participant's home to ask them about the potential causes of discordance. Before interviewing each participant, the research assistants checked the original SSI to determine how the respondent had described the location (e.g., “aunt's house” or “internet cabin”) or the GIS maps to locate a nearby reference point that might help the participant identify each discordant location (e.g., 2 blocks from market). Research assistants (nurses and biologists) were native Iquitos residents who received specific training on all steps of the interview process to ensure they were aware of sensitive issues they might encounter both when gathering initial SSI information, as well as while following up with discordant locations.

### Ethics Approval

Participants were given a 24–48 hour period to decide whether to participate or not in the study. For children, verbal assent of the minor and written consent of the parent or caretaker were required, whereas for adults, a written consent was required. After GPS data collection, a strict protocol for storage (in a secure MySQL database) and management was followed. The procedures for enrollment of participants and GPS data management were approved by the Institutional Review Boards (IRB) of the University of California at Davis (2007.15244), Emory University (IRB9162) and Tulane University through an inter-institutional IRB agreement with the United States Naval Medical Research Center Unit No. 6 (NAMRU-6). The NAMRU-6 IRB, located in Peru, also reviewed and approved the study (NMRCD 2007.0007). This IRB functions as a Peruvian IRB and is registered with the Peruvian Regulatory Agency for Clinical Trials with the number RCEI-78.

## Results

More than half of the 160 enrolled participants were females (58.5%) (Table 1). The lower number of males was due to the difficulty in finding them at home during regular interviewing hours. Recruitment was stratified by age; the age range sampled was 7 to 74 years. Recruitment varied across age groups (range of 25–46 per age group), with 7–18 year olds accounting for 28% of the tracked individuals (Table 1). Although not perfectly balanced among sexes and age groups, the recruited population represents a large and diverse demographic sample of the local population.

**Figure 1:**
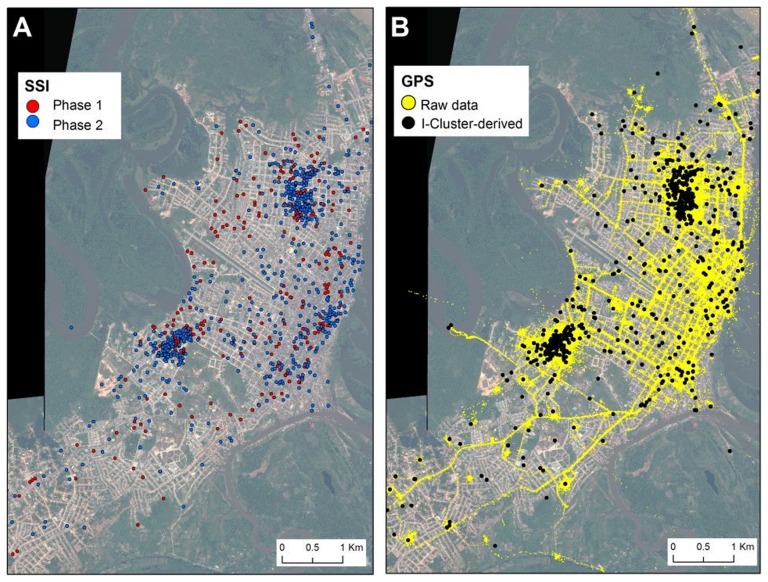
Locations inferred by (A) semi-structured interviews (SSI) and (B) GPS units. (A) Spatial distribution of all locations reported as visited by 160 participants during a 14-day period. (B) Raw GPS tracks (yellow points) and locations inferred after the application of a data-reduction algorithm (black dots) that assigns each track to a specific location code in the Iquitos GIS.

Of the 2,566 locations identified by SSI and/or i-Cluster algorithm, 14.3% were concurrently identified by both (i.e., concordant). SSI identified 2.3 times more locations than the i-Cluster algorithm, with residential (42.5%), commercial (26.4%) and educational (10.8%) spaces accounting for the highest degree of concordance between methods (Table 2). A total of 2,047 places were reported in the SSI as visited by all participants over the 14-day tracking period (of these 2047 places mentioned, 1057 were unique places, see Table 2). Most (96.7%) places were located within the urban and peri-urban areas of Iquitos (Figure 1A). Participants reported visiting a median (Q1–Q3) of 12 (9–16) places over the 14-day period, with the number of places not differing significantly between sexes (Wilcoxon rank sum test with continuity correction, W = 3140.5, *P* = 0.89). The most commonly reported location types on SSI that were not visualized using the i-Cluster algorithm (considering 1609 locations with land-use information) were commercial locations (34.2%) followed by residential (22.1%) and recreational (17.0%) locations (Table 2). The i-Cluster algorithm identified a total of 886 places as visited by participants while tracked (716 unique locations); 98.7% of which were found within the urban and peri-urban areas of Iquitos (Figure 1B). A significantly lower median (Q1–Q3) number of places per participant was registered by the i-Cluster algorithm in comparison to the SSI (7, 4.0–10.0; W = 11990, *P*<0.001). Residential spaces represented 58.6% of the 454 i-Cluster-identified locations with land-use information that were not reported on the SSI, followed by commercial (11.4%), educational (4.2%), and recreational (3.5%) locations (Table 2). Locations with highest percentage of concordance (i.e., per type of location, the number of concordant sites divided by the total number of sites obtained for that type of location through SSI and/or GPS) were educational settings (24%), followed by residential (19%), other (18%), and religious or market spaces (both at 13%).

**Table 2:** Comparison of number of “concordant” locations identified by semi-structured interviews and GPS from both study phases.

Type of location	Concordant % (n)	SSI+ GPS –a % (n)	GPS+ SSI-b % (n)	Total % (n)
Residential	42.5 (156)	22.1 (372)	58.6 (304)	32.4 (832)
Market/Shops	26.4 (97)	34.2 (575)	11.4 (59)	28.5 (731)
Recreational	10.1 (37)	17.0 (286)	3.5 (18)	13.3 (341)
Educational	10.8 (40)	6.3 (105)	4.2 (22)	6.5 (167)
Public Bldg.	0.8 (3)	4.1 (69)	1.7 (9)	3.2 (81)
Health	2.5 (9)	5.0 (84)	1.9 (10)	4.0 (103)
Church/religious	2.5 (9)	3.2 (53)	1.0 (5)	2.6 (67)
Cemetery	0.5 (2)	1.4 (23)	0	1.0 (25)
Lodging	0	0.7 (12)	1.9 (10)	0.9 (22)
Others	2.7 (10)	1.8 (30)	3.3 (17)	2.2 (57)
Missing land-use information	1.1 (4)	4.2 (71)	12.5 (65)	4.1 (106)
**TOTAL**	**367**	**1680**	**519**	**2566**

Concordance between methods occurred when both methods identified the same location as visited by the same participant. A clustering algorithm was used to summarize raw GPS points into specific locations.

a Locations identified on the SSI, but not on the GPS.

b Locations identified on the GPS (using a clustering algorithm), but not on the SSI.

**Figure 2:**
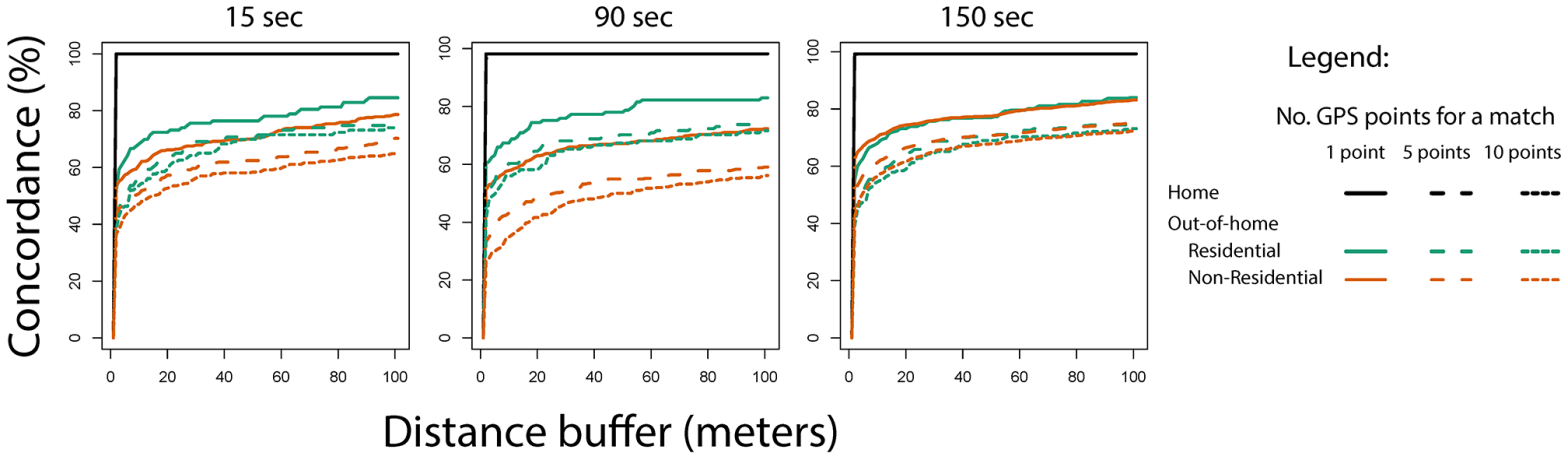
Concordance between SSI locations and raw GPS positions at different distance buffer thresholds, GPS data collection frequencies, and number of GPS points. Concordance was expressed as the percentage of locations for which a SSI-GPS match was found.

When the SSI-reported locations were compared to the raw GPS data (Figure 1), differences in the concordance between methods were observed based on the location type, distance threshold selected, GPS collection frequency and number of GPS points considered to define a visit (Figure 2). Both GPS and SSI had perfect concordance in identifying each participant's home (see Figure 2) at either combination of collection frequency, distance or number of points. There was more concordance for residential sites than non-residential sites at 15 and 90 seconds collection frequency; this difference was minimal at 150 seconds (Figure 2). Not depicted due to the small numbers in each category, there was much variation in concordance when examining by type of location. For example, when examining specific categories such as schools, “other” (ports, storage buildings, empty lots) and lodging places (i.e., rustic “hostels” for visitors from outside Iquitos, or couples might go for a few hours) there was a concordance of 80–100% between methods, whereas other residential places (i.e., friends' or relatives' homes), commercial locations (i.e., shops, markets) and religious buildings (i.e., churches) showed a concordance of 50–80%.

As distance from the SSI reported location increased, the concordance between SSI and raw GPS data increased, independently of the type of location (Figure 2). When at least one raw GPS point was considered (solid lines in Figure 2), concordance between methods was highest at up to 20 meters from each location. Beyond that distance, no dramatic increases in concordance were observed. There was less concordance when we restricted our analysis to 5 GPS points (broken lines) or 10 points (finely broken lines), but the pattern was similar to the line created when 1 point was considered a match. Interestingly, increasing frequency of GPS data collection from 150 to 15 seconds was not associated with a proportional increase in concordance between SSI and GPS (Figure 2). Battery power loss observed at 15 second collection frequency may help explain such results: of the 508 GPS exchanges performed, 56 (11%) of GPS units programmed to collect data every 15 seconds had issues due to battery loss at the time of data download in comparison to 2% (9/379) for GPS units programmed to collect data every 150 sec.

**Figure 3:**
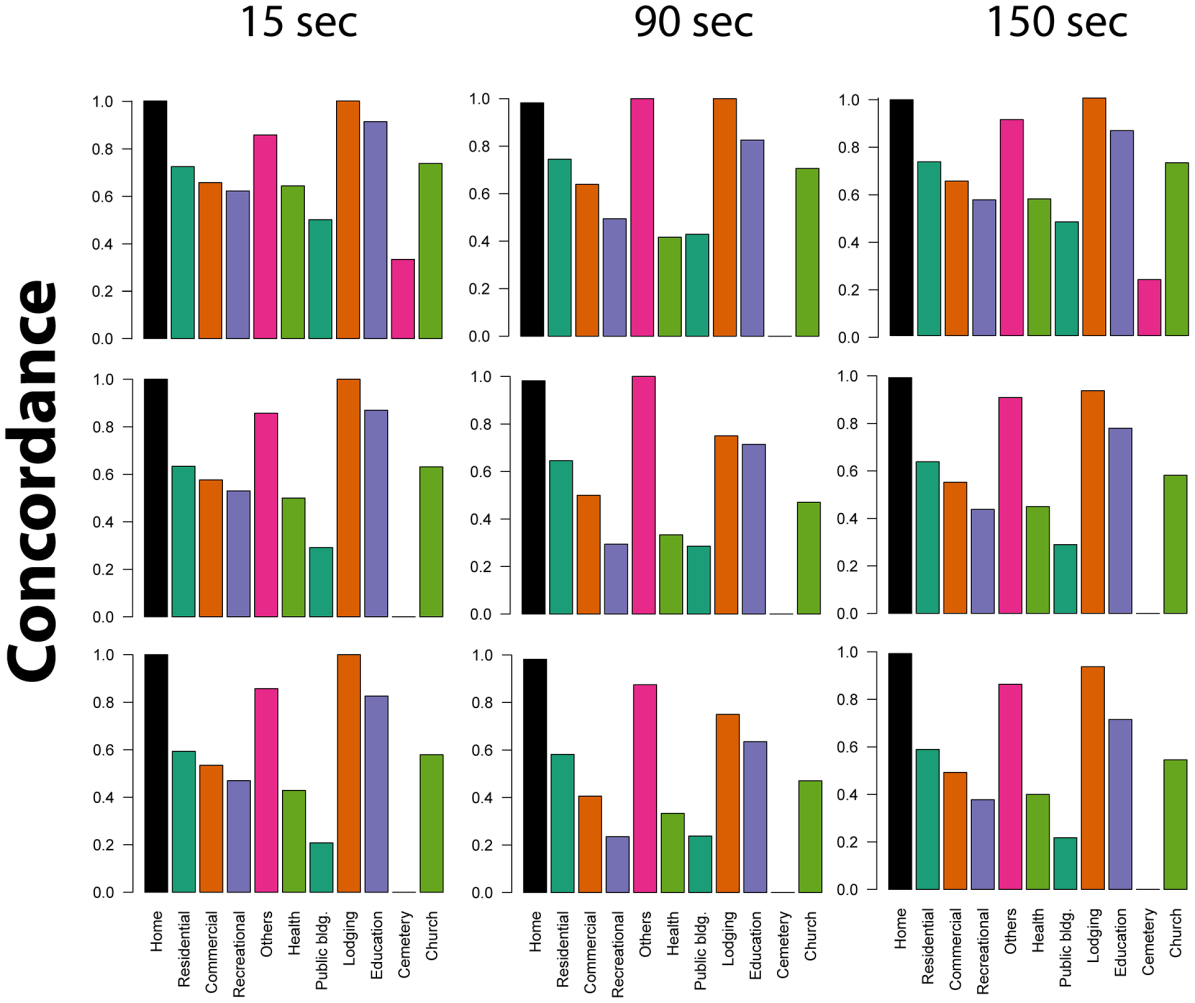
Concordance between SSI locations and raw GPS positions at 20 meters from a SSI location. Concordance is expressed as the proportion of locations for which a SSI-GPS match was found. Panels show values for different location types, combinations of GPS data collection frequencies (15, 90 and 150 seconds) and number of GPS points used to define a visit (1, 5 and 10 points).

At 20 meters from each SSI location, and when 1 GPS point was considered to define a match, overall concordance averaged 72.6% (SD: 20.7%) for 15 seconds, 65.8% (30.8%) for 90 seconds and 70.3% (23.3%) for 150 seconds (Figure 3). When ten points were required to define a match, concordance was reduced to 59.1% (31.6%), 54.3% (31.0%), and 55.7% (30.7%) respectively (Figure 3). Cemeteries, public buildings, recreational areas and health centers were the location types that consistently showed the lowest concordance values (Figure 3). Increasing the data collection frequency from 15 to 150 seconds did not translate into significant variation in the concordance between SSI and GPS across all location types (average [min-max] variation across locations, 2.3% [0.7%–9%]) (Figure 3).

In comparison to using the raw GPS points (Figure 2), the i-Cluster algorithm evidenced much higher discordance rates for all location types (Table 2). However, this method allowed identifying a total of 519 locations not mentioned in the SSI and not able to be inferred when the raw GPS positions were visualized (Table 2).

**Figure 4:**
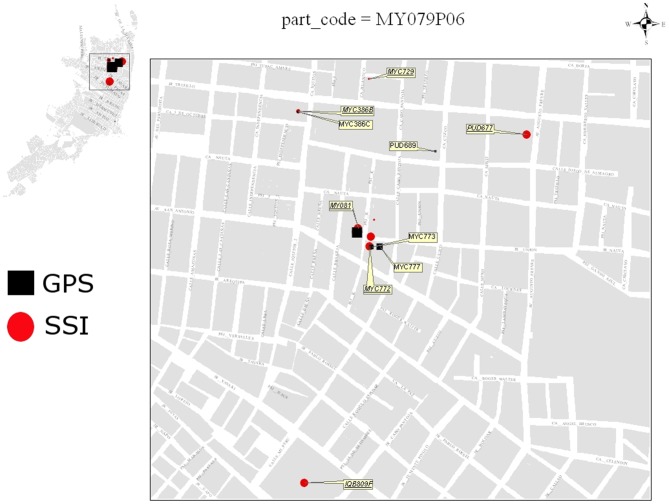
Sample map to interview participants about possible causes of discordance between GPS-derived vs. semi-structured interview locations. Given both types of locations were joined to the Iquitos GIS, the lot code was provided to ease identification of locations in the database. Size of points was proportional to reported or calculated time spent at each location. Inset of map shows locations within the city of Iquitos. GPS-derived locations were obtained using a clustering algorithm.

In Phase 2, with the subset of 101 participants, we further explored the possible causes of discordance between GPS and SSI. Specifically, within 2–3 days of administering the SSI, we used GIS to develop maps identifying “discordant” SSI and i-Cluster locations (Figure 4) (i.e., locations that were only mentioned in the SSI or only visualized using the GPS data). These maps were used when probing participants about possible causes of discordance. In this phase, regarding locations identified on the SSI, but not detected by GPS (total of 656 locations, Table 3), the most common response to questions about the discordance was an affirmation that these locations *had* been visited (35.8%) – they could not explain the discordance. The second most common response was that units had “seemed to be turned off” (30%). Indeed, GPS units initially deployed could accidentally be turned off, so respondents who noticed the lack of a flashing blue light inferred correctly. Once this problem was reported, we programmed GPS units to not allow them to be turned off manually, reducing this problem half-way through this study. Other explanations for the discordance included those who admitted forgetting to take units to some locations (12.5%; i.e., rushing out and simply forgetting), not wearing the GPS units to locations that were near their house (3.4%) or to locations where they might get stolen (3.5%), and leaving units home to recharge (4.6%). A small percentage (4.7%) affirmed having the GPS unit in some locations, but questioned whether the placement of the GPS unit in their purse might have impeded the signal.

**Table 3:** Reasons for discordance given by participants between locations from semi-structured interviews (SSI) and GPS data, from Phase 2 (n = 101).

Reason given for discordance	Number of locations (%)
**Location on SSI, but not on GPS (SSI+GPS−)**	**Total (locations): 656**
Says used GPS, no explanation for missing point	235 (35.8)
Says used GPS, but unit might have been off	197 (30.0)
Admits did not use GPS: rushed out and forgot	82 (12.5)
Says used GPS, but describes possible “barrier” (i.e., unit in purse, under a lot of clothes)	31 (4.7)
Admits did not use GPS, but no explanation given	33 (5.0)
Admits did not use GPS: it was recharging at home	30 (4.6)
Admits did not use GPS: concerned about GPS safety (not getting it stolen)	23 (3.5)
Admits did not use GPS: was going to a location near house	22 (3.4)
Admits did not use GPS: concerned about personal safety if wearing it in this location	3 (0.5)
**Location on GPS, but not on SSI (GPS+SSI−)**	**Total: 204**
Simply forgot to mention on SSI	78 (38.2)
Location on GPS was en route to another place	45 (22.1)
Forgot to mention on SSI because location not part of regular routine	31 (15.2)
Did not think to mention on SSI because location was outdoors	27 (13.2)
Doesn't remember being there	12 (5.9)
Embarrassed to mention on SSI	6 (2.9)
GPS was used by someone else in household	5 (2.5)
**Technical failures: no discordance**	**Total: 75**
Problem in merging methods	43 (57.3)
GPS marked location next door	32 (42.7)

A clustering algorithm was used to summarize raw GPS points into specific locations.

Regarding locations identified on the GPS unit but not mentioned in the SSI (204 locations, Table 3), the most common response was that they simply forgot to mention it in the SSI (38.2%), and a few made the additional observation that they had forgotten this location because it was not part of the regular routine (15.2%). Some locations were not mentioned (until probed directly about them) because they were either transient or en route to another location (22.1%; i.e., a path always taken, a bus stop) or because they were outdoors (13.2%; i.e., outdoor food kiosk). After further examination of the reasons for discordance between SSI and GPS, we identified 75 locations as being affected by technical failures in generating the maps (the locations were not properly mapped or marked the location next door, 57.3% and 42.7% respectively, and hence were incorrectly considered discordant at the time of interview).

## Discussion

GPS technology is increasingly used in behavioral research. Its use has moved beyond feasibility tests [15], [35], [37], [38] to the actual use of GPS-enabled devices (often coupled with other sensors such as accelerometers, air pollution sensors or cameras) in studies quantifying various aspects of human mobility and spatial behavior [7], [10], [11]. As the technology continues to be embraced by researchers across disciplines, it is easy to assume that due to the wealth and resolution of the data it provides, some might consider GPS data to be a “gold standard” for mobility research and a replacement of classic survey instruments [35]. By performing a field validation study tracking 160 individuals, we assessed both the limitations and possibilities of GPS technology for mobility research, and provided evidence of multiple sources of error/uncertainty that can affect quality of data in comparison to survey methods. It is important to mention here that based on our experience, we would expect different results with different GPS units, different SSI and other methods of data analysis.

Under perfect conditions of satellite geometry and signal strength, GPS provides very accurate information about the position (latitude, longitude, elevation, time of day) of any stationary object on earth. Wearable GPS devices provide all the essential pieces of information to reconstruct and quantify human movement: positions associated to places visited, time stamp for each potential visit, and routes followed to connect visits. Given technical (e.g., signal noise, multipath errors, signal obstruction inside buildings, battery life) and human behavioral limitations (e.g., compliance of use, individuals forgetting to take or charge units), GPS signals are prone to error and estimates of mobility parameters that they generate are considered uncertain. Signal processing algorithms have been developed to reduce such errors and improve interpretation of complex data [39]–[44]. In our study, the application of a signal clustering algorithm (i-Cluster) allowed identifying locations where individuals spent their time, but also added significant uncertainty by flagging locations transiently visited (e.g., a bus stop; 35.3%). Such errors were the main contributor to the 85.7% discordance between methods observed when i-Cluster inferred locations were considered. Because most research describing automated algorithms rely on single (or few) days of data or low sample sizes [39]–[44], the errors found by our study are a likely outcome of the type of error those algorithms may encounter if applied within the same context. Our results can be used as a guide for the development of improved and more accurate methods for GPS location extraction and human movement quantification.

An interesting finding was that higher GPS collection frequencies (e.g., 15 seconds) were not associated with a proportional and significant increase in concordance between methods. Issues of battery life, not securing the “off” option at the start of the study (remedied quickly), and compliance of participants in charging the units compromised the quality of data collected. Similar issues were observed across multiple studies quantifying physical activity [17], [35]. Implementing GPS-enabled smart-phones could have reduced the issue of battery loss, because there is more motivation for individuals to charge the phones overnight and to use them during the day. Because Iquitos is slowly making its transition into smart-phone technologies, different issues were pointed out by a subset of 10 participants when asked about the possibility of using GPS-phones instead of data-loggers: (a) older individuals were intimidated by the technology and by the possibility of having the units stolen (the latter was a concern shared by individuals across all age groups), and (b) school age children mentioned they are not allowed to take phones to elementary or high school or locations where their phones could be taken by older children [7]. When cell-phones can be properly deployed they can provide valuable information. For instance, in Canada a study comparing GPS data collected by cell-phones and self-reported surveys reported (using rudimentary indices of concordance such as convex hulls and kernel density estimations) that 75% of questionnaire-reported activity locations were located within 400 meters of an activity location recorded on the GPS track [26]. In weighting the possibility of adopting novel technologies, consideration of cultural and local concerns will be key for both GPS and SSI instruments [13], [16], [18].

Turning the large amounts of raw GPS positional data into meaningful locations individuals visited is another challenge. Unprocessed raw GPS data can be used to either describe zones or areas in which individuals spend their time or to assess the accuracy of the GPS in identifying precise locations against information provided by another method (i.e., locations identified by SSI). In our study we implemented a simple algorithm based on an agglomerative clustering method (i-Cluster) to identify locations visited by individuals carrying a GPS unit. Our analysis shows that the algorithm presents low levels of sensitivity and specificity in identifying places reported as visited by participants. This poor performance could be due to: (a) the algorithm's limited ability to account for changes in accuracy of the GPS signal or to the occurrence of intermittent positions as a consequence of GPS signal loss and (b) the fact that not all reported locations were actually visited by participants while tracked. More complex methods of location extraction that account for signal errors, such as hierarchical dynamic Bayesian network models [39], [41], [44], are being currently developed and are viewed as a promising means of reducing the uncertainty associated with the identification of locations visited by participants [39], [44]. Once those methods are validated, their integration into health research applications will increase our ability to accurately infer the location of potential infectious disease exposure areas.

Classic methods (surveys, diaries) have long been considered too limited to quantify behavior due to marked heterogeneities in the ability of individuals to recall the locations they visit, interviewer error, behavior changes and issues associated to privacy [35]. By working with the local community, addressing potential cultural barriers and concerns and adapting the language of interviews, we developed a culturally-sensitive SSI to quantify movement (and potential exposure to dengue). Our comparative analysis shows that, for a 14-day recall period, interviews provide accurate estimates of the locations visited by people (of a total of 892 locations for which we investigated causes of discordance, only 109 [12.2%] were visited and not reported). The SSI not only identified places, but also characterized the context of visits (i.e., grandmother's house), information impossible to obtain directly from GPS. SSI data entry and processing are much more straightforward and faster than of GPS: (a) maps with marked locations were digitized in the Iquitos GIS and each premise reported as visited was assigned a location code and (b) the location code was then linked to the database containing all the SSI information. We concluded that a validated survey instrument that can be adapted to different contexts can be used to understand the role of human mobility in infectious disease dynamics.

We encountered several limitations in our study design. Although our sample size was relatively large, the low numbers of participants assigned to each age group precluded statistical tests to look at different causes of discordance. Given that we needed to obtain results quickly to ask participants about possible causes of discordance, we relied on a single GPS data reduction algorithm (i-Cluster). As observed on the survey (Table 3), most of the discordant records occurred due to this algorithm providing false positive or negative results. Since the time this study was performed, new and more sophisticated methods to process GPS data have been developed [39]–[44], as well as more accurate and less error-prone GPS units have likely become available. Future research will involve performing comparative studies to quantify sensitivity/specificity as well as applicability to specific study questions. Also, we considered that our concordance estimates could be, in part, dependent on size and placement of houses in Iquitos. An average household in this city measures 5 m in width, which is within the mean error of a GPS (5–10 m). This could explain the high percent (∼60%) of residences identified by GPS that were not reported on the SSI. Thus, accuracy in identifying locations is not only dependent on the factors explained above, but also on key attributes of the urban landscape (e.g., household size, prevailing building material, density of high-rise buildings, vegetation cover). We did not test for differences in the SSI results of participants with more contact with our research team (i.e., those with more frequent GPS exchanges due to differing data collection times) compared to those with minimal contact. We do not expect differences, however, because contact was focused on the GPS exchange and SSI questions about their movement and activities were only asked at the end of the 14 day period. None of the participants, therefore, had an advantage over others regarding the types of questions they would be asked. We also did not estimate nor compare the cost and technical expertise to apply and process by these methods. Both the GPS and SSI capture very complex data. GPS data is in digital form, but needs to be processed. SSI data needs to be verified (i.e., in our study, someone might go to a location described to geocode the location), entered and mapped. There were costs associated to purchasing GPS units (∼$49/unit), training personnel to set and distribute units, downloading and analyzing the GPS data. Similarly, there were costs associated to developing, refining and improving the SSI, training personnel to apply it, and entering the data in a GIS system. Ultimately, decisions regarding using an SSI or GPS units in a study depend strongly on the study question and the urban context, because both SSI and GPS can provide different but equally valuable information that need to be carefully weighted at the planning stage.

For infectious diseases in general, and vector-borne diseases in particular, the need to tie potential exposure to specific locales requires the retrospective investigation of multiple routes of pathogen transmission. Survey instruments like the one we developed in this study not only provide accurate information of places visited, but can also be used to retrospectively infer the likely location where infection occurred [5]. This need to tie exposure to a specific place(s) has limited the use of GPS technology in infectious disease research, but GPS technology could be used in prospective movement studies or in studies obtaining information provided by phone companies. As observed in our study, once locations are identified, the raw GPS positions can be analyzed to quantify temporal patterns of mobility (days and times a person visits such locations, regularity of visits, overlap with other tracked individuals) and to accurately quantify routines and movement of a large segment of a population. This way, key information about mobility and behavior can be inferred and used to parameterize mathematical models that allow better forecasting of disease transmission or design policies targeting activities or segments of the population at greatest risk.

No gold standard exists for obtaining and analyzing human mobility data, instead different errors may occur with different methods. Despite the continually improving accuracy available with GPS, barriers persist, including: behavioral aspects (i.e., people remembering to use the unit), technical aspects (i.e., accuracy of 5–10 meters in a location with houses averaging 5 meters width), and analytical aspects (i.e., differences in concordance based on method of analyzing complex data as reported in this article). The SSI is not a gold standard either. Even with the possible drawback of more locations reported than true (i.e., false positives), compared to GPS units, the SSI provided more true locations, more context about locations, and data were easier to process and analyze. For our study, in which we needed to identify locations retrospectively for possible exposure to dengue virus, the SSI was the only choice because of the logistical and financial difficulty of fitting GPS units on a large sample and, even if that had been possible, being able to quickly identify locations recently visited within a short enough time frame to initiate our possible exposure investigations. For now, SSI remains the most comprehensive method to identify such locations.

## Supporting Information

Checklist S1 **STROBE checklist.**
(DOC)
