# Supplementary material for: Strengths and Weaknesses of Global Positioning System (GPS) Data-Loggers and Semi-structured Interviews for Capturing Fine-scale Human Mobility: Findings from Iquitos, Peru
Source: PLoS Negl Trop Dis. 2014 Jun 12;8(6):e2888. doi: 10.1371/journal.pntd.0002888 (PMC4055589; doi:10.1371/journal.pntd.0002888)
Supplement: Checklist S1 — STROBE checklist. (DOC) [file pntd.0002888.s001.doc]

STROBE Statement—Checklist of items

**Strengths and weaknesses of Global Positioning System (GPS) data-loggers and semi-structured interviews for capturing fine-scale human mobility: Findings from Iquitos, Peru**

|  | Item No | Recommendation |
| --- | --- | --- |
| **Title and abstract** | 1 | (*a*) Indicate the study’s design with a commonly used term in the title or the abstract  ***“Observational study” is included in the abstract.*** |
|  |  | (*b*) Provide in the abstract an informative and balanced summary of what was done and what was found  ***This has been done.*** |
| Introduction | | |
| Background/rationale | 2 | Explain the scientific background and rationale for the investigation being reported  ***This is done in the introduction. Namely, after describing increasing use of GPS-devices for research on human mobility, we describe some of the barriers to using these devices and alternative methods used to measure human mobility.*** |
| Objectives | 3 | State specific objectives, including any prespecified hypotheses  ***As described in the last paragraph of the introduction: “As part of a larger study investigating risk for dengue (a human disease caused by a mosquito transmitted virus) in Iquitos, Peru, we simultaneously implemented two methods to capture fine-scale human mobility patterns: GPS data-loggers and semi-structured interviews (SSI)…*** ***The main goal of this observational study was to compare and contrast the information obtained through these two methods to assess the issues affecting data quality, and identify strengths and weaknesses of each approach. We used two methods to analyze GPS data, and compared GPS results obtained via both methods with the results from the SSI.”*** |
| Methods | | |
| Study design | 4 | Present key elements of study design early in the paper  ***A brief description of our design was included in the abstract. A detailed description of our design was included in the section entitled “Recruitment, Participants and Study Design”*** |
| Setting | 5 | Describe the setting, locations, and relevant dates, including periods of recruitment, exposure, follow-up, and data collection  ***The first paragraph of the methods section describes the study setting in detail, plus overall dates of the study; more detail is included later in that section.*** |
| Participants | 6 | (*a*) Give the eligibility criteria, and the sources and methods of selection of participants. Describe methods of follow-up  ***This is done in methods, under the subheading “Recruitment, Participants and Study Design”. We also refer to Table 1 at this point for a demographic description of the participants.*** |
|  |  | (*b*) For matched studies, give matching criteria and number of exposed and unexposed  ***Not applicable.*** |
| Variables | 7 | Clearly define all outcomes, exposures, predictors, potential confounders, and effect modifiers. Give diagnostic criteria, if applicable  ***We describe the instruments that we compare in this analysis in detail under the subheading “Instruments”. We also carefully describe how we processed data from the GPS units and SSI in the section “Data Processing and Analysis”.*** |
| Data sources/ measurement | 8* | For each variable of interest, give sources of data and details of methods of assessment (measurement). Describe comparability of assessment methods if there is more than one group  ***For this article, this information is included under “Instruments” and “Data Processing and Analysis”.*** |
| Bias | 9 | Describe any efforts to address potential sources of bias  ***Issue of bias that could have interfered with the comparison of GPS vs. semi-structured interview data is discussed in a paragraph on limitations in the discussion.*** |
| Study size | 10 | Explain how the study size was arrived at.  ***A sentence on our sample size ends the section on “Recruitment, Participants and Study Design”. It states: “Our sample size was sufficient for a descriptive analysis and was limited due to intense participant follow-up for ~20 days; i.e., recruiting and consenting, distributing GPS units, exchanging charged GPS units and collecting ones losing power, interviewing participants with SSI at day 14, geocoding locations immediately, inputting all data from GPS and SSI to overlay in a GIS, returning to participants for follow up interview. Considering these complexities, participant recruitment was limited to what was logistically feasible for our field teams.”*** |
| Quantitative variables | 11 | Explain how quantitative variables were handled in the analyses. If applicable, describe which groupings were chosen and why  ***Information about how data is processed from both the GPS and semi-structured interviews, and then compared, is described in detail in “Data Processing and Analysis”.*** |
| Statistical methods | 12 | (*a*) Describe all statistical methods, including those used to control for confounding  ***The section with subheading “Data Processing and Analysis” describes how GPS data was processed, and methods used to consider “concordance” of data between semi-structured interviews and GPS units.*** |
|  |  | (*b*) Describe any methods used to examine subgroups and interactions  ***Not applicable.*** |
|  |  | (*c*) Explain how missing data were addressed  ***Not applicable.*** |
|  |  | (*d*) If applicable, explain how loss to follow-up was addressed  ***Not applicable.*** |
|  |  | (*e*) Describe any sensitivity analyses  ***Not applicable.*** |
| Results | | |
| Participants | 13* | (a) Report numbers of individuals at each stage of study—eg numbers potentially eligible, examined for eligibility, confirmed eligible, included in the study, completing follow-up, and analysed  ***Participant numbers are reported in table 1. Due to our design (intense follow up with 160 participants in short time frame), we did not have any loss to follow up. Using purposive sampling, we recruited 160 individuals to participate and the only criteria for exclusion was if they were going to be outside of Iquitos for more than a day in the following 14 days, as described in Methods section under “Recruitment, Participants and Study Design”.*** |
|  |  | (b) Give reasons for non-participation at each stage  ***We intended to recruit more men, but they were harder to find at their homes during interview times, as explained in the first two sentences of the results section.*** |
|  |  | (c) Consider use of a flow diagram  ***It was considered, but we did not feel it added much in this article.*** |
| Descriptive data | 14* | (a) Give characteristics of study participants (eg demographic, clinical, social) and information on exposures and potential confounders  ***Demographic information about participants included in Table 1.*** |
|  |  | (b) Indicate number of participants with missing data for each variable of interest  ***Not applicable.*** |
|  |  | (c) Summarise follow-up time (eg, average and total amount)  ***This is summarized in the methods section and was completed as planned in the study (i.e., we did not have to alter the procedures).*** |
| Outcome data | 15* | Report numbers of outcome events or summary measures over time  ***Done as applicable for this article in results section.*** |
| Main results | 16 | (*a*) Give unadjusted estimates and, if applicable, confounder-adjusted estimates and their precision (eg, 95% confidence interval). Make clear which confounders were adjusted for and why they were included  ***Not applicable.*** |
|  |  | (*b*) Report category boundaries when continuous variables were categorized  ***Not applicable.*** |
|  |  | (*c*) If relevant, consider translating estimates of relative risk into absolute risk for a meaningful time period  ***Not applicable.*** |
| Other analyses | 17 | Report other analyses done—eg analyses of subgroups and interactions, and sensitivity analyses  ***All analyses conducted are reported in the results section.*** |
| Discussion | | |
| Key results | 18 | Summarise key results with reference to study objectives  ***Summary of key results is included in the first paragraph of the discussion section, and our concluding paragraph refers to our study objectives as well.*** |
| Limitations | 19 | Discuss limitations of the study, taking into account sources of potential bias or imprecision. Discuss both direction and magnitude of any potential bias  ***Limitations (as well as potential bias) are described in the discussion section, in the paragraph starting with “We encountered several limitations in our study design…”*** |
| Interpretation | 20 | Give a cautious overall interpretation of results considering objectives, limitations, multiplicity of analyses, results from similar studies, and other relevant evidence  ***This is done throughout discussion, but also summarized in concluding paragraph.*** |
| Generalisability | 21 | Discuss the generalisability (external validity) of the study results  ***Though the word “generalizability” is not explicitly stated, the whole discussion section focuses on the strengths and weaknesses of two methods: GPS units that can be used anywhere in the world (and if anything, using them in places with addresses that are not all geocoded, like Iquitos, might present additional barriers) and semi-structured interviews. We are very careful to state that although we found that the SSI identified more locations in our analysis, it is an instrument that needs to be locally/culturally adapted and validated.*** |
| Other information | | |
| Funding | 22 | Give the source of funding and the role of the funders for the present study and, if applicable, for the original study on which the present article is based.  ***This is included in the financial statement.*** |
